# Effects of pharyngeal electrical stimulation on swallowing performance

**DOI:** 10.1371/journal.pone.0190608

**Published:** 2018-01-02

**Authors:** Ryosuke Takeishi, Jin Magara, Masahiro Watanabe, Takanori Tsujimura, Hirokazu Hayashi, Kazuhiro Hori, Makoto Inoue

**Affiliations:** 1 Division of Dysphagia Rehabilitation, Niigata University Graduate School of Medical and Dental Sciences, 2–5274 Gakkocho-dori, Chuo-ku, Niigata, Japan; 2 Division of Comprehensive Prosthodontics, Niigata University Graduate School of Medical and Dental Sciences, 2–5274 Gakkocho-dori, Chuo-ku, Niigata, Japan; Hangzhou Normal University, CHINA

## Abstract

Pharyngeal electrical stimulation (PEStim) has been found to facilitate voluntary swallowing. This study investigated how PEStim contributed to modulation of swallowing function in 15 healthy humans. In the involuntary swallowing test, water was injected onto the pharynx at 0.05 ml/s and the onset latency of the first swallow was measured. In the voluntary swallowing test, subjects swallowed their own saliva as quickly as possible for 30 s and the number of swallows was counted. Voluntary and involuntary swallowing was evaluated before (baseline), immediately after, and every 10 min after 10-min PEStim for 60 min. A voluntary swallowing test with simultaneous 30-s PEStim was also conducted before and 60 min after 10-min PEStim. The number of voluntary swallows with simultaneous PEStim significantly increased over 60 min after 10-min PEStim compared with the baseline. The onset latency of the first swallow in the involuntary swallowing test was not affected by 10-min PEStim. The results suggest that PEStim may have a long-term facilitatory effect on the initiation of voluntary swallowing in healthy humans, but not on peripherally-evoked swallowing. The physiological implications of this modulation are discussed.

## Introduction

The mechanism of swallowing involves complex sensorimotor neural components. The complexity of swallowing may be explained by the fact that swallowing has several functions, including propelling the food bolus from the oral cavity into the stomach through the pharynx and the esophagus, and protecting the upper respiratory tract by cleaning the larynx and pharynx; hence preventing choking or aspiration of secretions or food [1–4]. In addition, to complete normal swallowing movements, more than 25 pairs of related muscles in the orofacial, pharyngeal, laryngeal, and esophageal regions must be activated bilaterally and in coordination [1–3].

Underlying motor patterns of swallowing are programmed by the so-called central pattern generator (CPG) in the medulla oblongata, and both the peripheral and central inputs into the CPG can trigger swallowing [2, 3]. In other words, swallowing can be initiated either involuntarily or voluntarily. Peripherally-evoked swallowing can be initiated by mechanical or chemical stimulation in the oropharynx or larynx. Sensory regions that elicit pharyngeal swallowing include the soft palate, uvula, dorsal tongue surface, faucial pillars, dorsal pharyngeal wall, pharyngeal surface of the epiglottis, and the glossoepiglottic sinus [5–9]. Stimulation of the superior laryngeal nerve (SLN) and the pharyngeal branch of the glossopharyngeal nerve innervating the hypopharyngeal and laryngeal regions are known to be effective in triggering repetitive pharyngeal swallowing, even in anesthetized animals [10–12]. Because initiation of the swallowing reflex is not interrupted after ablation of the cortex, peripheral inputs may be effective enough to initiate swallowing [11]. One can therefore hypothesize that, as in animals, certain patterns of electrical stimulation applied to these nerves or regions can activate and/or facilitate the activation of the swallowing CPG in humans.

Our previous studies showed that repetitive pharyngeal electrical stimulation (PEStim) facilitated initiation of voluntary rapid swallowing in conscious humans [13, 14]. This result was expected because the stimulated areas were innervated by the SLN or the pharyngeal branch of the glossopharyngeal nerve, and therefore the swallowing CPG was activated by PEStim. However, facilitation of voluntary swallowing by this method may not be comparable to that of involuntary or reflexively evoked swallowing. Takatsuji et al. [15] reported that continuous PEStim failed to elicit repetitive involuntary swallowing, although the first swallow was successfully evoked following stimulation. Contrary to this report, it is known that continuous SLN electrical stimulation can readily evoke a repetitive swallowing reflex [2]. Possible reasons for the discrepancy between the animal and human studies described above are: (1) a species difference; (2) a difference in the stimulus conditions, in that surface stimulation is not effective in fully activating the neural network in humans; or (3) a difference in the experimental condition, in that cortical activity might inhibit the neural circuit of swallowing in either the cortical/subcortical areas or the brainstem in the conscious condition.

Previous studies, some of which used subthreshold electrical stimulation applied to the peripheral regions, demonstrated that the stimulation produced long-lasting changes in swallowing-associated motor cortical excitability. Power et al. [16] showed that after 10-min palatal electrical stimulation, all swallowing measures, including oral transit time, swallowing response time (defined as the time interval between the presentation of the bolus at the hypopharynx and laryngeal elevation), airway closure duration, and cricopharyngeal opening time, remained unaffected. Contrary to this, Fraser et al. [17] and Jayansekeran et al. [18] showed that, after 10-min PEStim, pharyngeal transit time, swallowing response time, and aspiration were significantly improved in acute stroke patients. These conflicting results suggest that it has not been fully clarified how PEStim contributes to changes in swallowing behavior, particularly the initiation of the swallowing movement.

Transcranial magnetic stimulation (TMS) of the brain causes the peripheral muscles to produce neuroelectrical signals known as motor-evoked potentials (MEPs). Fraser et al. and Power et al. showed that a 10-min session of pharyngeal and palatal electrical stimulation increased corticobulbar excitability 60 min after the stimulation ended, as measured using TMS [16, 17, 19]. The long-term effect was also observed in brain imaging data; Fraser et al. [17] reported that the cortical blood oxygenation level-dependent fMRI signal showed greater bilateral functional activation within the sensorimotor cortex (BA 3/4) 60 min after 10-min PEStim compared with no PEStim. Interestingly, the ideal stimulation frequency for facilitation of MEPs differed between pharyngeal and palatal stimulation. For pharyngeal stimulation, the facilitatory effect was noted at 5 Hz and 75% of the maximum tolerated stimulation amplitude [17, 19], and at 0.2 Hz and 75% of maximum tolerated sensation for palatal stimulation [16]. The difference in the stimulus frequency may be explained by that in the stimulus site. The oral mucosa is innervated by the glossopharyngeal and trigeminal nerves. The trigeminal nerve includes rich mechano-sensory fibers conducting not only innocuous, but also noxious, sensations. Electrical stimulation at high frequency may activate these fibers, resulting in inhibition of the swallowing neural network, i.e., swallowing initiation [20, 21].

In any case, changes in the neural network involved in swallowing may affect related behavior. Because swallowing can be initiated either involuntarily or voluntarily, we should consider how experimental interventions such as electrical stimulation changes swallowing function. The onset latency of swallowing evoked by peripheral stimulation seems to be an appropriate way to evaluate involuntary swallowing function, because most swallowing reflexes can be evoked involuntarily by pharyngeal or laryngeal stimulation. However, because initiation of voluntary swallowing essentially needs activation of higher centers including the sensorimotor cortex, the number of swallowing events evoked voluntarily can be one of the methods that evaluates voluntary swallowing function. Thus, to investigate the mechanism of action of PEStim further, we studied the effect of 10-min PEStim on swallowing function by: (1) measuring the onset latency of the first involuntary swallow during water infusion at a very slow rate, which represents the excitability of the swallowing reflex arc; and (2) measuring the number of voluntary swallows for 30 s, which represents the corticobulbar excitability for the initiation of voluntary swallowing.

## Materials and methods

### Participants and ethical approval

Fifteen healthy male adults (mean age ± SD: 26.7 ± 5.7 years; age range: 22–37 years) participated in the study. Informed consent was obtained from all participants, and no subject had a history of alimentary disease, pulmonary disease, neurological disease, musculoskeletal disorders, speech disorders, voice problems, or masticating or swallowing problems. The experiments were approved by the Ethics Committee of the Faculty of Dentistry, Niigata University (25-R33-11-25).

### Experimental conditions

To reduce a possible effect of circadian variations or the environment on swallowing performance, experiments were performed at the same time of day in an air-conditioned room, where room temperature was maintained at 20–24°C and humidity at 40–70%. Subjects were asked to refrain from eating, drinking, smoking, and brushing their teeth for at least 60 min before the experiment. The subjects were seated comfortably and remained upright throughout the study.

To monitor swallowing events, electromyographic (EMG) and electroglottographic (EGG) activity was recorded as performed in previous studies [22–26]. Bipolar surface EMG electrodes (WEB-1000; Nihon Kohden, Tokyo, Japan) were attached to the skin over the anterior surface of the digastric muscle on the left side, and EMG signals were detected in the suprahyoid muscle group. The signals were filtered and amplified (low cut, 30 Hz and high cut, 2 kHz) (WEB-1000; Nihon Kohden). Bipolar surface EGG electrodes were positioned on either side of the thyroid cartilage and the signals were amplified (EGG-D200; Laryngograph, London, UK). Amplified EMG and EGG signals were stored through an interface board (PowerLab; ADInstruments, Colorado Springs, CO, USA) on a personal computer. The sampling rate was 10 kHz. Data were analyzed using the PowerLab software package (LabChart6; ADInstruments).

For PEStim, catheter electrodes (TK210-107b; Unique Medical Co., Ltd, Tokyo, Japan) were developed. The catheter had two platinum ring electrodes for electrical stimulation, and was inserted transnasally. The distal electrode was 3 mm from the tip of the catheter, with a distance of 13 mm between the electrodes. The stimulation site was on the lateral wall of the laryngopharynx, at the level of the pyriform sinus, and was confirmed by videoendoscopy. The portion of the catheter electrode for PEStim outside the naris was taped. A minimum of 5 min was allowed for the subjects to become accustomed to the catheter. Bipolar surface electrical stimulation (1 ms pulse duration; 5 Hz) was delivered through cables connected to an electrical stimulator (SEN3401, Nihon Kohden). To determine the intensity of the stimulus, the current was increased by 0.1 mA every 5 s. Once stimulus thresholds for perception (STper) and tolerability (STtol) were determined by the subjects’ cues, the stimulus modality was determined as 75% of the maximum tolerated intensity, calculated as STper + 0.75 (STtol − STper), at a frequency of 5 Hz and a pulse duration of 1 ms, according to the method of a previous study [17].

**Fig 1:**
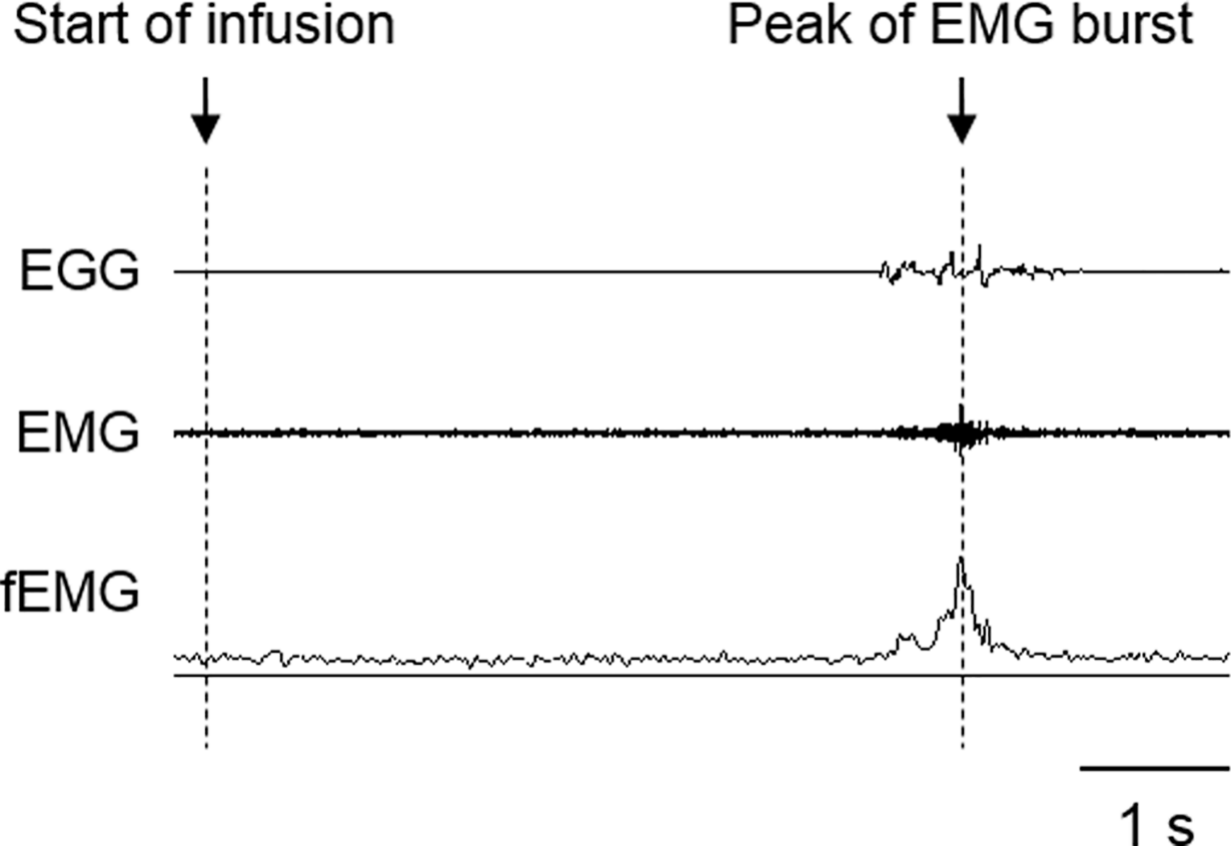
Measurement of onset latency of the first swallow in the involuntary swallowing test. A suprahyoid EMG and EGG were recorded with an infusion of distilled water at 0.05 ml/s into the pharynx. The time duration between the start of the infusion and the peak of the filtered EMG waveform during involuntary swallowing was measured. A swallowing event was also identified by EGG signals. EMG, electromyography; fEMG, filtered (rectified and smoothed) EMG; EGG, electroglottography.

Involuntary and voluntary swallowing tests were introduced in our previous studies [13, 14]. For the involuntary swallowing test, thickened water (Oishi-mizu; Asahi Soft Drinks, Ibaraki, Japan) was prepared with 1% thickening agent (Toromi Up Perfect; The Nissin Oilio Group, Ltd., Tokyo, Japan). Following setup of the recording and stimulating devices, a thin tube (2.7 mm outer diameter; NIPRO, Osaka, Japan) was inserted into the pharynx transnasally. The tip of the tube was positioned at the posterior wall of the midpharynx and was confirmed by videoendoscopy. The portion of the tube outside the naris was taped. The subject was asked to swallow his own saliva prior to recording to clear the saliva from the oral and/or pharyngeal cavity. The liquid was then delivered through the tube using an infusion pump (KDS-100; Muromachi, Tokyo, Japan). The infusion was started at the end of the expiratory phase. To minimize the mechanical effect of the infused solution, it was infused at a very slow rate (0.05 ml/s) until the first involuntary swallow was evoked. The subjects were blinded to the start of water infusion. The onset latency of the first involuntary swallow was measured (Fig 1).

**Fig 2:**
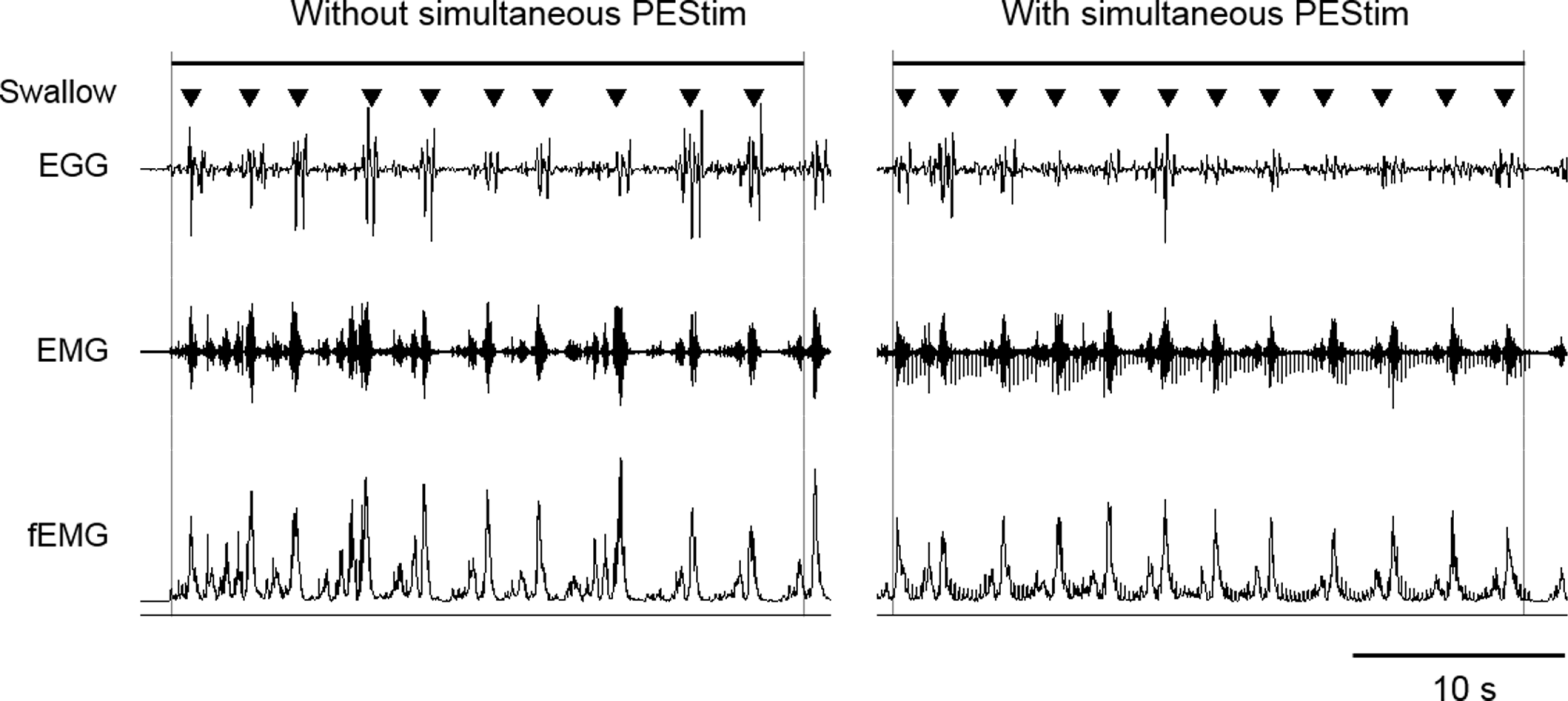
Example of EGG and suprahyoid EMG recordings in the voluntary swallowing test with and without simultaneous PEStim. A swallowing event was identified by EGG and EMG bursts and indicated by a closed triangle. In this case, the number of swallows over 30 s increased with PEStim from 10 to 12. EMG, electromyography; EGG, electroglottography; PEStim, pharyngeal electrical stimulation.

In the voluntary swallowing test, subjects were instructed to engage in repetitive swallowing behavior as quickly as possible for 30 s and the number of swallows was counted (Fig 2). In our previous studies, we confirmed the high reproducibility of these values in each individual [13, 14].

### Data collection

**Fig 3:**
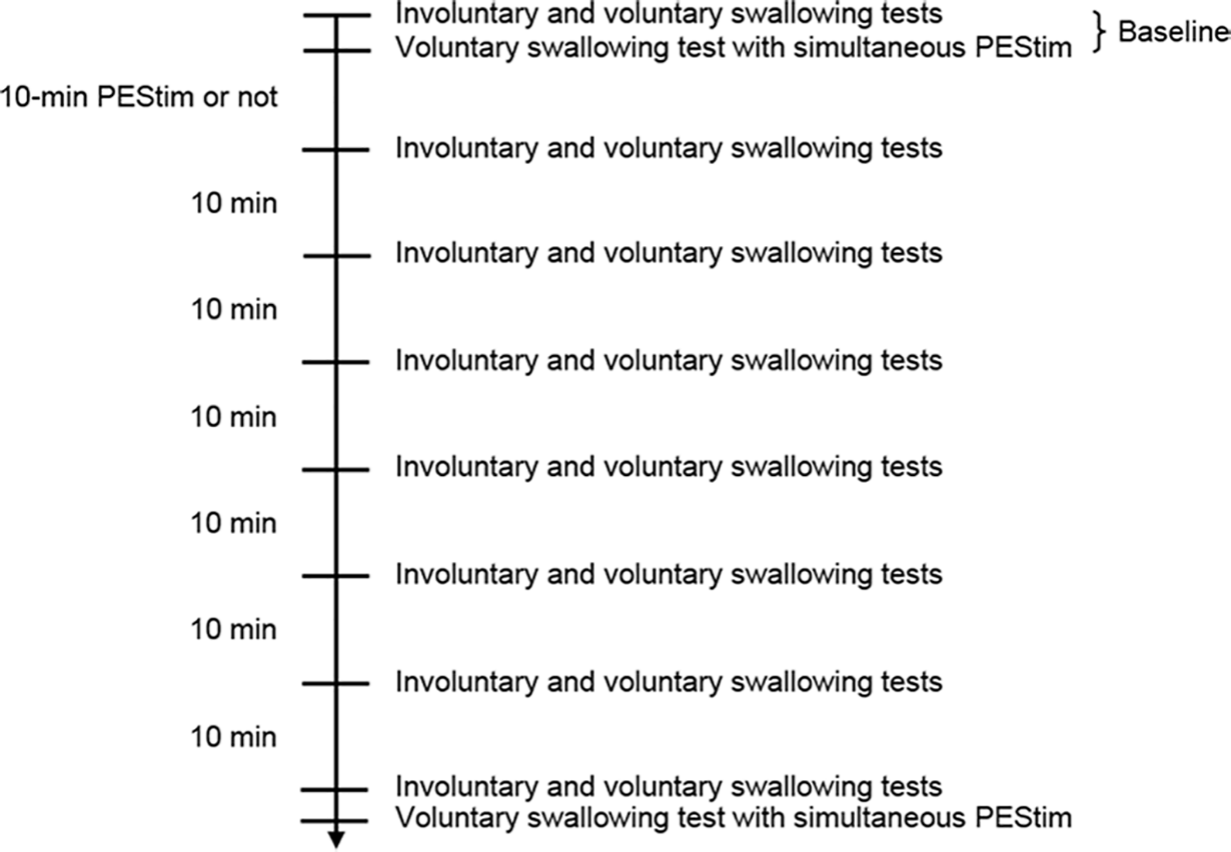
Experimental protocol of the study. Baseline, involuntary and voluntary swallowing tests were conducted followed by a voluntary swallowing test with simultaneous PEStim. Then 10-min PEStim was delivered to one group, but not the other group. Immediately afterwards and every 10 min up to 60 min, both involuntary and voluntary swallowing tests were performed in both groups. Finally, a voluntary swallowing test with simultaneous PEStim was performed. PEStim, pharyngeal electrical stimulation.

The participants were randomly divided into two groups so that each subject participated in the experiment on one day only. One group was provided with 10-min PEStim (stimulation group, n = 9) and the other was not (sham group, n = 6). The experimental protocol is shown in Fig 3. Firstly, baseline data were obtained from an involuntary swallowing test, voluntary swallowing test and voluntary swallowing test with PEStim. The time interval between the tests was set at 2 min. Following this, in the stimulation group, 10-min PEStim was applied with the same stimulus modality as before. During the 10-min PEStim, subjects were instructed to keep quiet, but there was no limit on spontaneous saliva swallowing. In the sham group, all the procedures were the same as for the stimulation group but the 10-min PEStim was not delivered. The involuntary swallowing test and voluntary swallowing test were performed immediately after the 10-min PEStim (or no stimulation in the sham group) and at 10-min intervals for 60 min (Fig 3). Finally, the voluntary swallowing test with simultaneous PEStim was performed. The voluntary swallowing test with simultaneous PEStim was performed only twice: before (baseline) and 60 min after the 10-min PEStim.

### Data analysis

Baseline data were compared between the groups with and without simultaneous PEStim in the voluntary swallowing test and between the stimulation and sham groups using a paired t-test.

In each group, the mean values of the onset latency of the first swallow in the involuntary swallowing test and the number of swallows in the voluntary swallowing test without simultaneous PEStim were compared at different times using one-way repeated-measures analysis of variance (ANOVA) and Dunnett’s test as post hoc analysis.

In the analysis of the number of swallows to evaluate the effect of 10-min PEStim on voluntary swallowing behaviors, data obtained from the voluntary swallowing test with and without simultaneous PEStim were compared using two-way repeated-measures ANOVA with two factors: time-point (baseline vs 60 min after 10-min PEStim) and intervention (with vs without simultaneous PEStim). Post hoc analysis using the Tukey test was performed if significant interactions between the factors were observed. The analysis was performed not only for the stimulation group, but also for the sham group.

Because there was no limit on spontaneous saliva swallowing during the 10-min PEStim in the stimulation group, it is possible that the number of spontaneous swallows varied among the subjects and may have affected the subsequent responses, such as the number of voluntary swallows and the onset latency of the first involuntary swallow after the 10-min PEStim. We therefore evaluated the relationship between the number of swallows evoked during the 10-min PEStim and changes in the number of swallows or the onset latency of the first involuntary swallow using linear regression analysis at two time points: immediately after and 60 min after the 10-min PEStim.

Tests for statistical differences and comparison tests were performed using statistical software (SigmaPlot 12; Systat Software Inc., San Jose, CA, USA). Statistical significance was set at P < 0.05. All values were expressed as mean ± SD.

## Results

### Baseline data

The threshold of the stimulus current for perceived and tolerated sensations varied among the 15 subjects at 1.1 ± 1.3 mA and 3.2 ± 2.5 mA, respectively. No subjects reported discomfort related to simultaneous or 10-min PEStim.

The mean onset latency of the first involuntary swallow was 6.58 ± 2.89 s (n = 15); 6.54 ± 2.26 s (n = 9) in the stimulation group and 6.64 ± 3.90 s (n = 6) in the sham group. There was no difference between the stimulation and sham groups.

The mean number of voluntary swallows was 7.3 ± 2.3 (n = 15) without simultaneous PEStim and 10.0 ± 3.2 (n = 15) with simultaneous PEStim; 7.8 ± 2.4 and 9.3 ± 2.6, respectively, in the stimulation group (n = 9) and 6.5 ± 2.2 and 11.0 ± 4.0, respectively, in the sham group (n = 6). As expected, there was a significant difference in the number of voluntary swallows between with and without simultaneous PEStim in all the groups (P < 0.05) while there was no difference between the stimulation and sham groups.

### Effects of 10-min PEStim on involuntary and voluntary swallows

**Fig 4:**
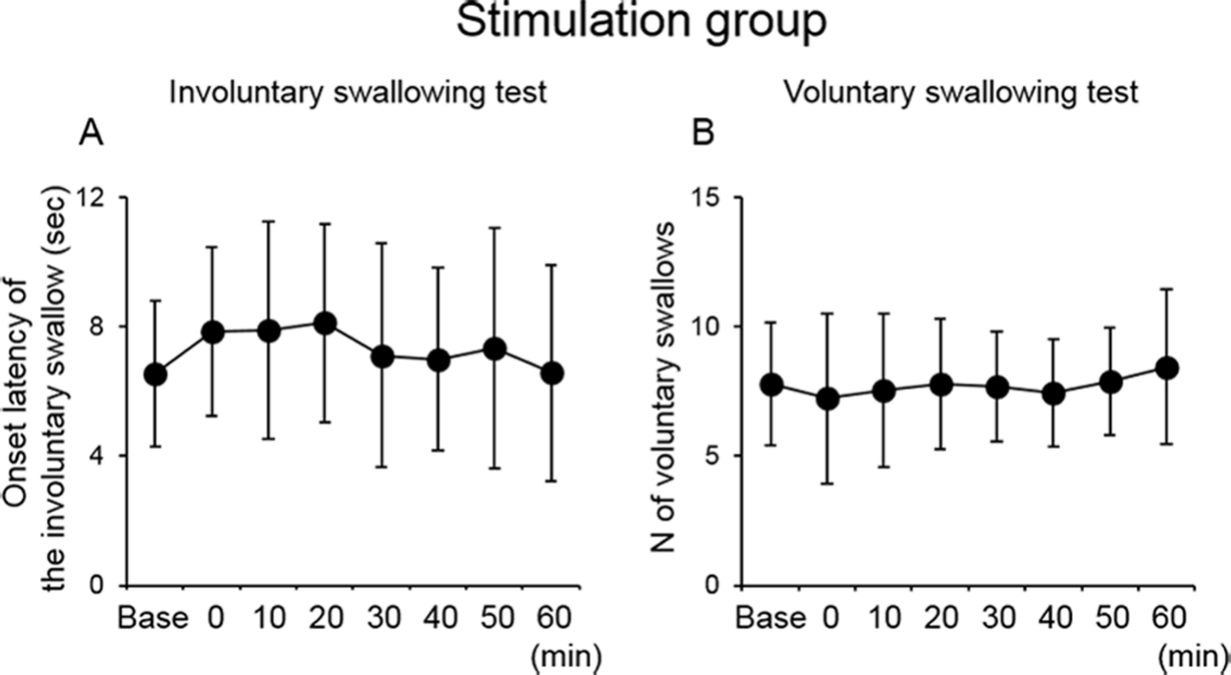
Effect of 10-min PEStim in the stimulation group. The onset latency of the first swallow in the involuntary swallowing test (A) and the number of swallows without simultaneous PEStim in the voluntary swallowing test (B) did not exhibit any significant changes, although the former was slightly longer immediately after 10-min PEStim, and the latter slightly decreased immediately after 10-min PEStim and gradually increased over 60 min. Base, baseline; PEStim, pharyngeal electrical stimulation.

**Fig 5:**
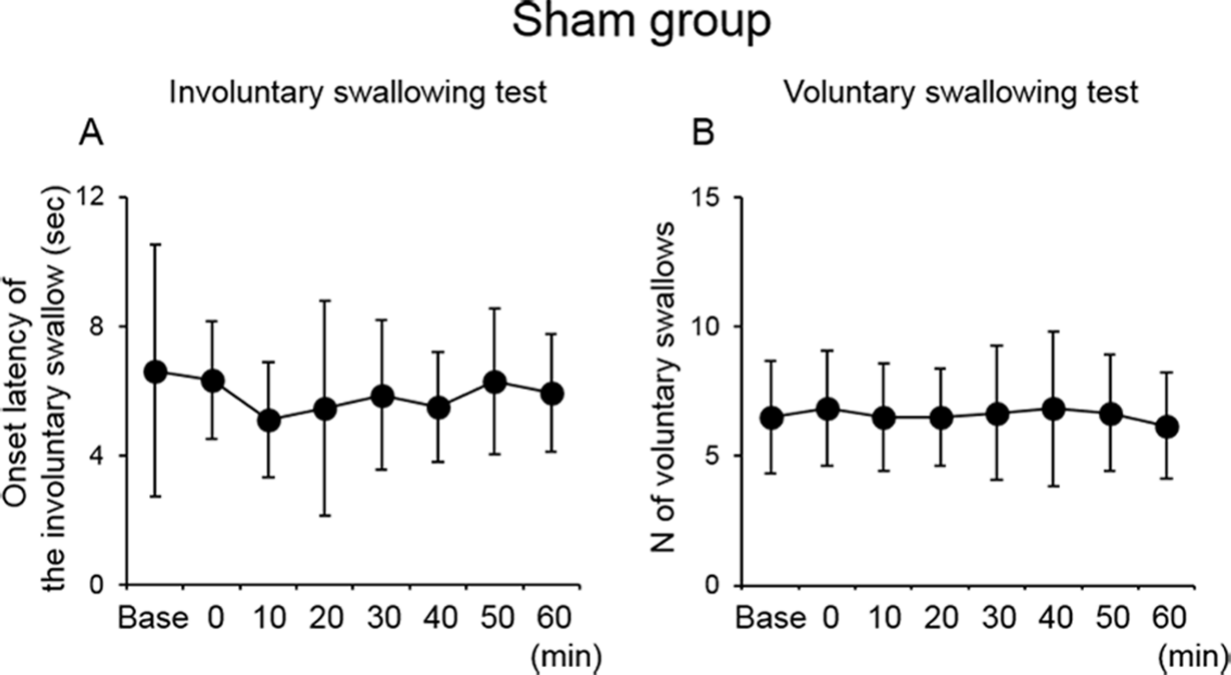
Effect of PEStim in the sham group. The onset latency of the first swallow in the involuntary swallowing test (A) and the number of swallows without simultaneous PEStim in the voluntary swallowing test (B) did not exhibit any significant changes. Base, baseline; PEStim, pharyngeal electrical stimulation.

Immediately after 10-min PEStim, the onset latency of the first involuntary swallow tended to be longer compared with the baseline and gradually decreased (Fig 4A). The number of voluntary swallows tended to decrease immediately after 10-min PEStim compared with the baseline (Fig 4B), although there was no significant difference in those values among the time points. In the sham group, there was no significant difference in the onset latency of the first swallow and the number of swallows without simultaneous PEStim over time (Fig 5A and 5B).

**Fig 6:**
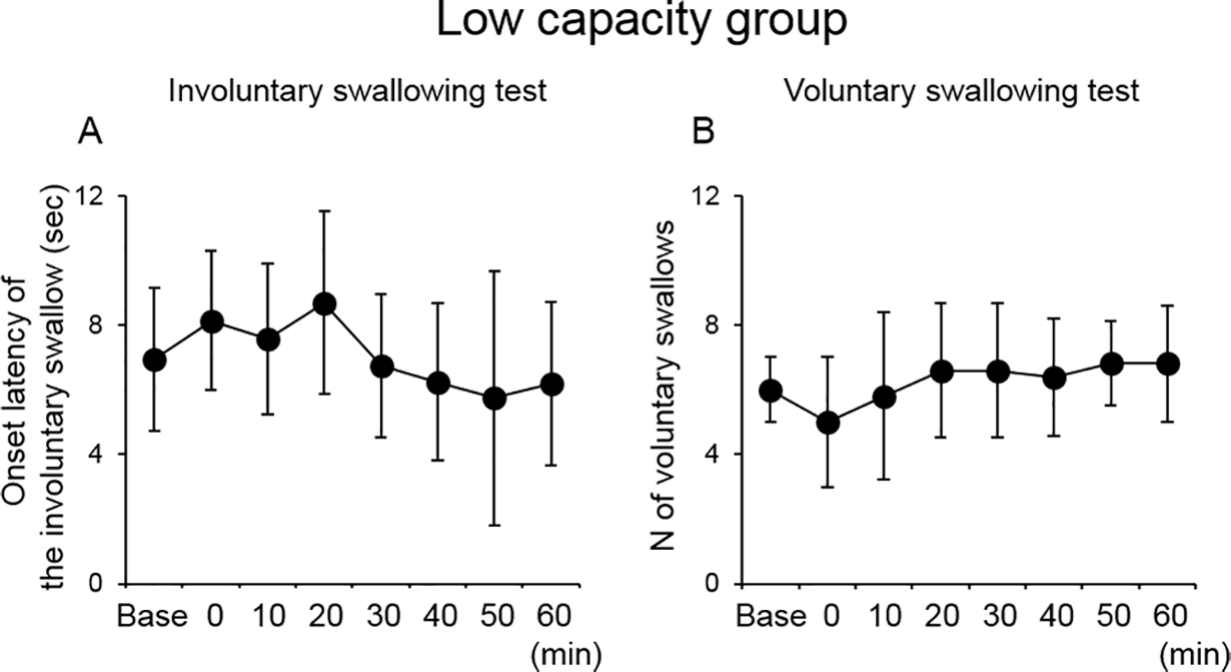
Effect of 10-min PEStim in the subject group whose swallowing capacity was low. The number of swallows without simultaneous PEStim in the voluntary swallowing test exhibited significant changes without any significant difference between the two mean values. Base, baseline; PEStim, pharyngeal electrical stimulation.

We suspected that 10-min PEStim was not strong enough to cause a change in the subsequent swallowing function, at least in healthy subjects. Therefore, this analysis was also performed for subjects (n = 6) for whom the onset latency of the first involuntary swallow was higher than the mean value (6.58 s) or the number of voluntary swallows was lower than the mean value (7.3 swallows) in the stimulation group. There was a significant difference in the number of voluntary swallows without simultaneous PEStim among the time points (P = 0.002) without any significant difference between the two mean values while there was no significant difference in the onset latency of the first swallow (Fig 6).

### Effects of 10-min PEStim on voluntary swallows with and without simultaneous PEStim

**Fig 7:**
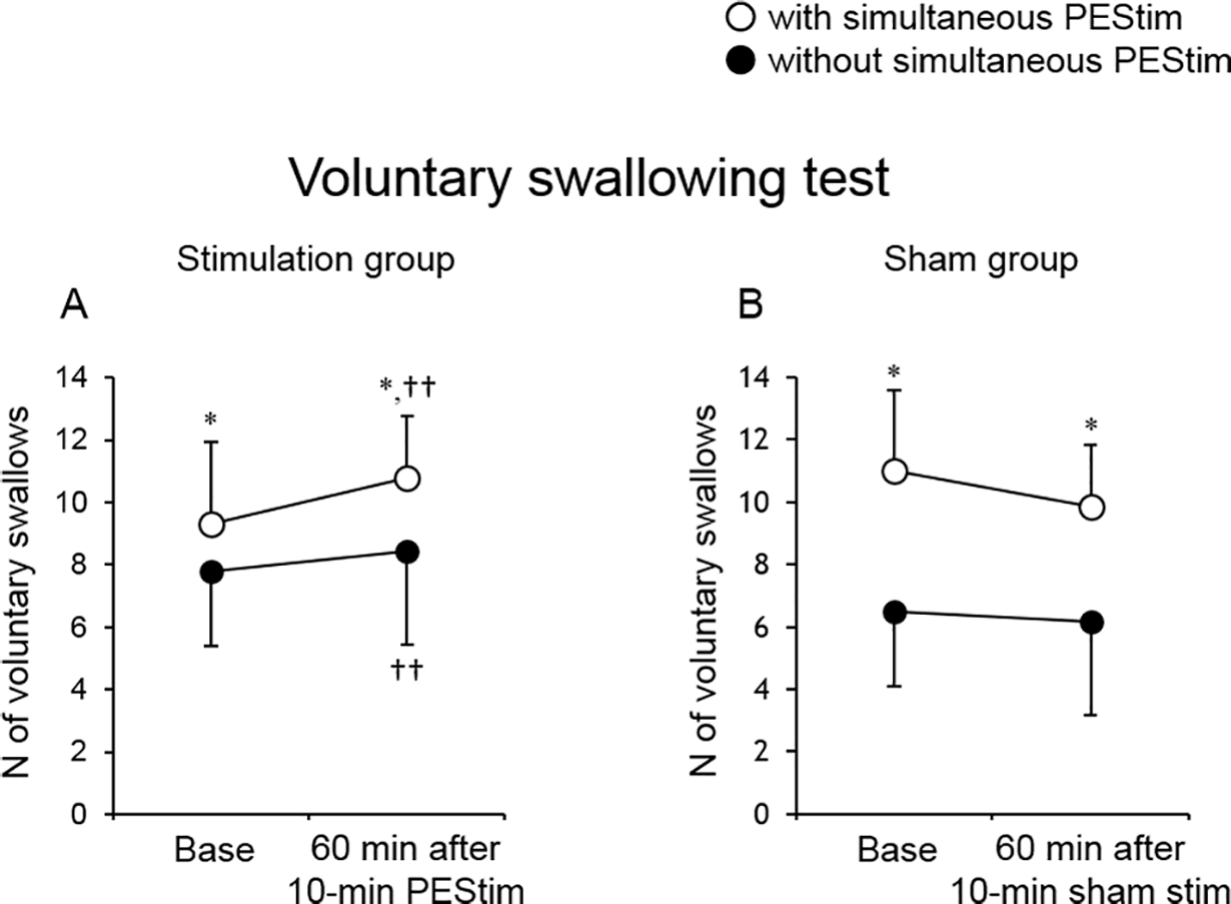
Effect of 10-min PEStim on voluntary swallowing. In the stimulation group (A), the mean number of swallows in the voluntary swallowing test with and without simultaneous PEStim was 9.3 ± 2.6 (n = 9) and 7.8 ± 2.4 (n = 9), respectively, at baseline and 10.8 ± 2.0 (n = 9) and 8.4 ± 3.0 (n = 9), respectively, 60 min after 10-min PEStim. Overall, these results show that 10-min PEStim had long-term facilitatory effects on subsequent voluntary swallowing initiation. In the sham group (B), the mean number of swallows in the voluntary swallowing test with and without simultaneous PEStim was 11.0 ± 4.0 (n = 6) and 6.5 ± 2.2 (n = 6) respectively at baseline, and 9.8 ± 3.0 (n = 6) and 6.2 ± 2.0 (n = 6) respectively at 60 min after 10-min PEStim. There was no difference in the number of voluntary swallows with and without simultaneous PEStim between the time points (baseline vs 60 min after 10-min PEStim). Base, baseline; PEStim, pharyngeal electrical stimulation. *P < 0.05 vs without simultaneous PEStim, ††P < 0.01 vs baseline.

The mean values of the number of voluntary swallows with and without simultaneous PEStim at baseline and 60 min after 10-min PEStim in the stimulation group are shown in Fig 7A. Two-way repeated-measures ANOVA comparing the number of voluntary swallows for time points with or without simultaneous PEStim revealed no significant interaction (degree of freedom (DF) = 1, sum of squares (SS) = 1.361, P = 0.357). Further analysis showed a significant difference between with and without simultaneous PEStim (DF = 1, SS = 34.028, P = 0.004) and between the two time-points (DF = 1, SS = 10.028, P = 0.016) at baseline and 60 min after 10-min PEStim. These results show that simultaneous PEStim may facilitate voluntary swallowing initiation and that 10-min PEStim may have a long-term facilitatory effect on subsequent voluntary swallowing initiation. This was not the case in the sham group (Fig 7B). Two-way repeated-measures ANOVA comparing the number of voluntary swallows at time points with or without simultaneous PEStim revealed no significant interaction (DF = 1, SS = 1.042, P = 0.601). Further analysis showed a significant difference between with and without simultaneous PEStim (DF = 1, SS = 100.042, P = 0.020) but not between the two time-points (DF = 1, SS = 3.375, P = 0.215) at baseline and 60 min after 10-min PEStim.

### Relationship between the number of swallows in 10-min PEStim and after-effects

The number of evoked swallows during 10-min PEStim varied widely, and the mean number of swallows was 33.4 ± 14.1 (n = 9), ranging from 12 to 55. Because it was possible that the number of evoked swallows during 10-min PEStim affected the subsequent changes in the initiation of involuntary and voluntary swallows, we evaluated the relationship between evoked swallows in 10-min PEStim. There was no significant correlation between the number of swallows and all the parameters analyzed (Table 1). The results suggest that facilitation of voluntary swallowing after 10-min PEStim was not directly related to the number of swallows evoked during 10-min PEStim.

**Table 1:** Relationship between the number of swallows evoked during 10-min PEStim and changes in swallowing function.

		DF	SS	F	P
Onset latency of the first involuntary swallow	0 min	1	448.128	2.721	0.143
	60 min	1	307.447	1.664	0.238
Number of voluntary swallows	0 min	1	33.335	0.149	0.711
	60 min	1	70.127	0.321	0.589

0 min data represent the difference in the values between the baseline and immediately after 10-min PEStim, and 60 min data represent the difference in the values between the baseline and 60 min after 10-min PEStim.

## Discussion

### Long-term effect of PEStim

One of the aims of the present study was to evaluate the long-term (60 min after 10-min PEStim) effect of PEStim on swallowing performance, including involuntary and voluntary swallowing. Changes in sensory inputs can produce persistent changes in the organization of sensory and motor areas of the cerebral cortex [27, 28]. Previous human and animal studies reported that a reduction in sensory feedback can induce changes in motor representation in the cerebral cortex [29–32]. Fraser et al. [17] demonstrated that changes in peripheral input can remodel human cortical motor organization in humans. They showed that cortical TMS-evoked motor potentials in the pharyngeal and esophageal muscles were highly dependent upon the frequency, intensity, and duration of PEStim, with 5 Hz for 10 min inducing stronger cortical activation. It can be assumed that the stimulus modality employed in the present study was appropriate for evaluation of long-term effects on swallowing performance.

We found that 10-min PEStim exerted a facilitatory effect on the voluntary swallowing function only with the assistance of simultaneous PEStim, suggesting that the normal swallowing function in healthy subjects was rarely changed just by the PEStim employed in our study. Numerous studies have reported long-term effects of PEStim in healthy subjects and dysphagic patients. In healthy subjects receiving PEStim, TMS-evoked potentials in the pharyngeal and esophageal muscles increased over time [17–19, 33, 34]. Additionally, at the same time, the number of brain sites evoking a response in the pharyngeal and esophageal muscles by TMS increased bilaterally in the cortex, and an increase in the areas of voxel activation was observed in the lateral sensorimotor cortex by fMRI [17]. Suntrup et al. [35] performed whole-head magnetoencephalography and observed a marked reduction in event-related desynchronization during voluntary swallowing in sensorimotor brain areas 45 min after PEStim. As with the brain imaging data, their study showed that volume per swallow and swallowing capacity significantly increased following PEStim. In dysphagic patients, electrophysiological data showed a marked increase in pharyngeal corticobulbar excitability and topographic representation in the undamaged hemisphere, and there was also an improvement in swallowing function, including shorter pharyngeal transit time, shorter onset latency of pharyngeal swallowing during voluntary swallowing, and decreased frequency of aspiration [17]. These results partly support the findings of the current study, in that the number of voluntary swallows with simultaneous PEStim increased at 60 min after 10-min PEStim, and the number of voluntary swallows without simultaneous PEStim was affected in subjects who had a relatively low capacity of swallowing function.

Although the current experiments did not address the mechanism of the sensory driven effects and why they were able to facilitate voluntary swallowing even in healthy subjects, it can be suggested that the effects on swallowing-related cortical excitability may be related to such phenomena as long-term potentiation. In fact, the time course of the facilitatory effect was similar to that seen in another study on the human motor cortex [36]. Nevertheless, the precise mechanisms for the modulation of the swallowing function obtained in the present study cannot be addressed in experiments on the human cortex, and should be addressed in further studies.

### Limitations

Several limitations affected the interpretation of the present findings. First, we recruited only 15 healthy male participants. Because the samples were small and the values varied widely among the subjects, we obtained the effect size and the required sample size of each parameter. The level of significance was set at P < 0.05, and the desired statistical power of the trial was set at 0.8. With the sample sizes of the groups set to be equal, the effect size was 0.47 for one-way ANOVA, and 0.47 for two-way ANOVA, both of which were considered medium effect. In future studies, we should evaluate the effect of PEStim on swallowing performance in young females as well as males to clarify the sex difference. Because subjects who exhibited a low capacity swallowing function tended to increase the number of voluntary swallows over time, future studies should include older participants or patients whose swallowing function is impaired. Finally, our findings that the voluntary swallowing function was affected by PEStim were determined only by counting the number of swallows. In our next study, we will combine the use of TMS to measure MEPs in the related muscles with our current methods to explain how MEPs are related to swallowing behavior.

## References

[1] Doty RW. Neural organization of deglutition Handbook of Physiology The Alimentary Canal. IV. Washington D. C.: Am Physiol Soc; 1968 p. 1861–902.

[2] Jean A. Brain stem control of swallowing: neuronal network and cellular mechanisms. Physiol Rev. 2001;81(2):929–69. doi: 10.1152/physrev.2001.81.2.929 11274347 10.1152/physrev.2001.81.2.929

[3] Miller AJ. Deglutition. Physiol Rev. 1982;62(1):129–84. doi: 10.1152/physrev.1982.62.1.129 7034008 10.1152/physrev.1982.62.1.129

[4] Nishino T. Swallowing as a protective reflex for the upper respiratory tract. Anesthesiology. 1993;79(3):588–601. Epub 1993/09/01. . 8363086 10.1097/00000542-199309000-00024

[5] Pommerenke WT. A study of the sensory areas eliciting the swallowing reflex. Am J Physiol. 1928;84:36–41.

[6] Sinclair WJ. Initiation of reflex swallowing from the naso- and oropharynx. Am J Physiol. 1970;218(4):956–60. Epub 1970/04/01. doi: 10.1152/ajplegacy.1970.218.4.956 . 5435428 10.1152/ajplegacy.1970.218.4.956

[7] Sinclair WJ. Role of the pharyngeal plexus in initiation of swallowing. Am J Physiol. 1971;221(5):1260–3. Epub 1971/11/01. doi: 10.1152/ajplegacy.1971.221.5.1260 . 5124270 10.1152/ajplegacy.1971.221.5.1260

[8] Storey AT. Laryngeal initiation of swallowing. Exp Neurol. 1968;20(3):359–65. Epub 1968/03/01. 0014-4886(68)90079-4 [pii]. . 5656849 10.1016/0014-4886(68)90079-4

[9] Storey AT. A functional analysis of sensory units innervating epiglottis and larynx. Exp Neurol. 1968;20(3):366–83. Epub 1968/03/01. 0014-4886(68)90080-0 [pii]. . 5656850 10.1016/0014-4886(68)90080-0

[10] Kitagawa J, Shingai T, Takahashi Y, Yamada Y. Pharyngeal branch of the glossopharyngeal nerve plays a major role in reflex swallowing from the pharynx. Am J Physiol Regul Integr Comp Physiol. 2002;282(5):R1342–7. Epub 2002/04/18. doi: 10.1152/ajpregu.00556.2001 . 11959674 10.1152/ajpregu.00556.2001

[11] Tsuji K, Tsujimura T, Magara J, Sakai S, Nakamura Y, Inoue M. Changes in the frequency of swallowing during electrical stimulation of superior laryngeal nerve in rats. Brain Res Bull. 2014;111C:53–61. Epub 2014/12/30. S0361-9230(14)00197-X [pii] doi: 10.1016/j.brainresbull.2014.12.008 . 25542096 10.1016/j.brainresbull.2014.12.008

[12] Tsujimura T, Udemgba C, Inoue M, Canning BJ. Laryngeal and tracheal afferent nerve stimulation evokes swallowing in anaesthetized guinea pigs. J Physiol. 2013;591(Pt 18):4667–79. Epub 2013/07/17. jphysiol.2013.256024 [pii] doi: 10.1113/jphysiol.2013.256024 . 23858010 10.1113/jphysiol.2013.256024 PMC3784206

[13] Aida S, Takeishi R, Magara J, Watanabe M, Ito K, Nakamura Y, et al Peripheral and central control of swallowing initiation in healthy humans. Physiol Behav. 2015;151:401–11. Epub 2015/08/09. S0031-9384(15)30058-5 [pii] doi: 10.1016/j.physbeh.2015.08.003 . 26253217 10.1016/j.physbeh.2015.08.003

[14] Tsukano H, Taniguchi H, Hori K, Tsujimura T, Nakamura Y, Inoue M. Individual-dependent effects of pharyngeal electrical stimulation on swallowing in healthy humans. Physiol Behav. 2012;106(2):218–23. Epub 2012/02/22. S0031-9384(12)00074-1 [pii] doi: 10.1016/j.physbeh.2012.02.007 . 22349483 10.1016/j.physbeh.2012.02.007

[15] Takatsuji H, Zakir HM, Mostafeezur RM, Saito I, Yamada Y, Yamamura K, et al Induction of the Swallowing Reflex by Electrical Stimulation of the Posterior Oropharyngeal Region in Awake Humans. Dysphagia. 2012;27(4):473–80. Epub 2012/01/31. doi: 10.1007/s00455-012-9393-1 . 22286211 10.1007/s00455-012-9393-1

[16] Power M, Fraser C, Hobson A, Rothwell JC, Mistry S, Nicholson DA, et al Changes in pharyngeal corticobulbar excitability and swallowing behavior after oral stimulation. Am J Physiol Gastrointest Liver Physiol. 2004;286(1):G45–50. Epub 2003/08/30. doi: 10.1152/ajpgi.00114.2003 00114.2003 [pii]. . 12946939 10.1152/ajpgi.00114.2003

[17] Fraser C, Power M, Hamdy S, Rothwell J, Hobday D, Hollander I, et al Driving plasticity in human adult motor cortex is associated with improved motor function after brain injury. Neuron. 2002;34(5):831–40. Epub 2002/06/14. S0896627302007055 [pii]. . 12062028 10.1016/s0896-6273(02)00705-5

[18] Jayasekeran V, Singh S, Tyrrell P, Michou E, Jefferson S, Mistry S, et al Adjunctive functional pharyngeal electrical stimulation reverses swallowing disability after brain lesions. Gastroenterology. 2010;138(5):1737–46. Epub 2010/02/09. S0016-5085(10)00161-7 [pii] doi: 10.1053/j.gastro.2010.01.052 . 20138037 10.1053/j.gastro.2010.01.052

[19] Fraser C, Rothwell J, Power M, Hobson A, Thompson D, Hamdy S. Differential changes in human pharyngoesophageal motor excitability induced by swallowing, pharyngeal stimulation, and anesthesia. Am J Physiol Gastrointest Liver Physiol. 2003;285(1):G137–44. Epub 2003/02/28. doi: 10.1152/ajpgi.00399.2002 00399.2002 [pii]. . 12606304 10.1152/ajpgi.00399.2002

[20] Tsujimura T, Kondo M, Kitagawa J, Tsuboi Y, Saito K, Tohara H, et al Involvement of ERK phosphorylation in brainstem neurons in modulation of swallowing reflex in rats. J Physiol. 2009;587(Pt 4):805–17. Epub 2009/01/07. jphysiol.2008.165324 [pii] doi: 10.1113/jphysiol.2008.165324 . 19124539 10.1113/jphysiol.2008.165324 PMC2669972

[21] Tsujimura T, Shinoda M, Honda K, Hitomi S, Kiyomoto M, Matsuura S, et al Organization of pERK-immunoreactive cells in trigeminal spinal nucleus caudalis, upper cervical cord, NTS and Pa5 following capsaicin injection into masticatory and swallowing-related muscles in rats. Brain Res. 2011;1417:45–54. Epub 2011/09/13. S0006-8993(11)01537-X [pii] doi: 10.1016/j.brainres.2011.08.032 . 21907330 10.1016/j.brainres.2011.08.032

[22] Nakamura Y, Hatakeyama A, Kitada Y, Tsujimura T, Taniguchi H, Inoue M. Effects of pharyngeal water stimulation on swallowing behaviors in healthy humans. Exp Brain Res. 2013;230(2):197–205. Epub 2013/07/17. doi: 10.1007/s00221-013-3641-y . 23857168 10.1007/s00221-013-3641-y

[23] Perlman AL, Grayhack JP. Use of the electroglottograph for measurement of temporal aspects of the swallow: preliminary observations. Dysphagia. 1991;6(2):88–93. Epub 1991/01/01. . 1935264 10.1007/BF02493485

[24] Schultz JL, Perlman AL, VanDaele DJ. Laryngeal movement, oropharyngeal pressure, and submental muscle contraction during swallowing. Arch Phys Med Rehabil. 1994;75(2):183–8. Epub 1994/02/01. 0003-9993(94)90393-X [pii]. . 8311675

[25] Taniguchi H, Tsukada T, Ootaki S, Yamada Y, Inoue M. Correspondence between food consistency and suprahyoid muscle activity, tongue pressure, and bolus transit times during the oropharyngeal phase of swallowing. J Appl Physiol. 2008;105(3):791–9. Epub 2008/06/17. 90485.2008 [pii] doi: 10.1152/japplphysiol.90485.2008 . 18556429 10.1152/japplphysiol.90485.2008

[26] Tsukada T, Taniguchi H, Ootaki S, Yamada Y, Inoue M. Effects of food texture and head posture on oropharyngeal swallowing. J Appl Physiol. 2009;106(6):1848–57. Epub 2009/03/28. 91295.2008 [pii] doi: 10.1152/japplphysiol.91295.2008 . 19325027 10.1152/japplphysiol.91295.2008

[27] Jenkins WM, Merzenich MM, Ochs MT, Allard T, Guic-Robles E. Functional reorganization of primary somatosensory cortex in adult owl monkeys after behaviorally controlled tactile stimulation. J Neurophysiol. 1990;63(1):82–104. Epub 1990/01/01. doi: 10.1152/jn.1990.63.1.82 . 2299388 10.1152/jn.1990.63.1.82

[28] Wang X, Merzenich MM, Sameshima K, Jenkins WM. Remodelling of hand representation in adult cortex determined by timing of tactile stimulation. Nature. 1995;378(6552):71–5. Epub 1995/11/02. doi: 10.1038/378071a0 . 7477291 10.1038/378071a0

[29] Brasil-Neto JP, Cohen LG, Pascual-Leone A, Jabir FK, Wall RT, Hallett M. Rapid reversible modulation of human motor outputs after transient deafferentation of the forearm: a study with transcranial magnetic stimulation. Neurology. 1992;42(7):1302–6. Epub 1992/07/01. . 1620338 10.1212/wnl.42.7.1302

[30] Brasil-Neto JP, Valls-Sole J, Pascual-Leone A, Cammarota A, Amassian VE, Cracco R, et al Rapid modulation of human cortical motor outputs following ischaemic nerve block. Brain. 1993;116 (Pt 3):511–25. Epub 1993/06/01. . 8513390 10.1093/brain/116.3.511

[31] Cohen LG, Brasil-Neto JP, Pascual-Leone A, Hallett M. Plasticity of cortical motor output organization following deafferentation, cerebral lesions, and skill acquisition. Adv Neurol. 1993;63:187–200. Epub 1993/01/01. . 8279304

[32] Sessle BJ, Yao D, Nishiura H, Yoshino K, Lee JC, Martin RE, et al Properties and plasticity of the primate somatosensory and motor cortex related to orofacial sensorimotor function. Clin Exp Pharmacol Physiol. 2005;32(1–2):109–14. Epub 2005/02/26. CEP4137 [pii] doi: 10.1111/j.1440-1681.2005.04137.x . 15730444 10.1111/j.1440-1681.2005.04137.x

[33] Hamdy S, Jilani S, Price V, Parker C, Hall N, Power M. Modulation of human swallowing behaviour by thermal and chemical stimulation in health and after brain injury. Neurogastroenterol Motil. 2003;15(1):69–77. Epub 2003/02/18. 390 [pii]. . 12588471 10.1046/j.1365-2982.2003.00390.x

[34] Hamdy S, Rothwell JC, Aziz Q, Singh KD, Thompson DG. Long-term reorganization of human motor cortex driven by short-term sensory stimulation. Nat Neurosci. 1998;1(1):64–8. Epub 1999/04/09. doi: 10.1038/264 . 10195111 10.1038/264

[35] Suntrup S, Teismann I, Wollbrink A, Winkels M, Warnecke T, Pantev C, et al Pharyngeal electrical stimulation can modulate swallowing in cortical processing and behavior—Magnetoencephalographic evidence. Neuroimage. 2014;104:117–24. Epub 2014/12/03. S1053-8119(14)00840-4 [pii] doi: 10.1016/j.neuroimage.2014.10.016 . 25451471 10.1016/j.neuroimage.2014.10.016

[36] Stefan K, Kunesch E, Cohen LG, Benecke R, Classen J. Induction of plasticity in the human motor cortex by paired associative stimulation. Brain. 2000;123 Pt 3:572–84. Epub 2000/02/25. . 10686179 10.1093/brain/123.3.572

